# Interaction and efficacy of Keigai-rengyo-to extract and acupuncture in male patients with acne vulgaris: A study protocol for a randomized controlled pilot trial

**DOI:** 10.1186/1745-6215-12-82

**Published:** 2011-03-19

**Authors:** Kyu Seok Kim, Yoon-Bum Kim

**Affiliations:** 1 Department of Ophthalmology & Otolaryngology & Dermatology, College of Oriental Medicine, Kyung Hee University, Seoul, Republic of Korea; 2 Department of Ophthalmology & Otolaryngology & Dermatology, College of Oriental Medicine, Kyung Hee University, 1 Hoegi-dong, Dongdaemun-gu, Seoul 130-701, Republic of Korea

## Abstract

**Background:**

In consideration of patients seeking to use traditional Chinese medicine, an evidence-based potentiality for safe and effective use of herbal medicine and acupuncture in treatment of acne vulgaris has been suggested. However, despite common use of a combination of herbal medicine and acupuncture in clinical practice, the current level of evidence is insufficient to draw a conclusion for an interaction and efficacy of herbal medicine and acupuncture. Therefore, considering these methodological flaws, this study was designed to assess the interaction and efficacy of an available herbal medicine, Keigai-rengyo-to extract (KRTE), and acupuncture for treatment of acne using the 2 × 2 factorial design and the feasibility of a large clinical trial.

**Methods/Design:**

A randomized, assessor single blinded, 2 × 2 factorial pilot trial will be conducted. Forty four participants with acne vulgaris will be randomized into one of four groups: waiting list group (WL), KRTE only group (KO), acupuncture only group (AO), and KRTE and acupuncture combined treatment group (KA). After randomization, a total of 8 sessions of acupuncture treatment will be performed twice a week in the AO- and KA groups, respectively. Patients in the KO- and KA groups will be prescribed KRTE 3 times a day at a dose of 7.4 g after meals for 4 weeks. The following outcome measurements will be used in examination of subjects: the mean percentage change and the count change of inflammatory and non-inflammatory acne lesions, the Skindex 29, visual analogue scale (VAS) and investigator global assessment (IGA) from baseline to the end of the trial.

**Trial Registration:**

The trial is registered with the Clinical Research Information Service (CRiS), Republic of Korea: KCT0000071.

## Background

Acne vulgaris is a common skin disease encountered in dermatology practice; it is caused by changes in pilosebaceous units [1,2]. It is common in adolescence and may proceed into adulthood, affecting roughly 33% of people between the ages of 15 and 44 years [3], or affecting nearly 80% of adolescents and young adults aged 11 to 30 years [4].

Acne vulgaris is a chronic dermatosis of the pilosebaceous follicle with four fundamental etiopathogenic factors: sebaceous hyperproduction, follicular hyperkeratinization, increase of *Propionibacterium acnes *colonization, and periglandular dermal inflammation [5]. Western medical treatment options generally target one or more of the factors implicated in these acne pathogeneses [6] with topical or systemic agents, phototherapy narrowband light (blue or red), and acne vaccines [7-9].

Despite this treatment for care of acne vulgaris, a substantial number of acne patients widely seek to use traditional Chinese medicine (TCM) due to a lack of response to western medical treatment [10] and their concern regarding side effects, such as burning, erythema, desquamation, pigmentation, xerosis, and chapped lips, among others [11-15].

Herbal medicine and acupuncture are two of the major tools of TCM therapy and have been frequently administered for treatment of acne symptoms in clinical practice worldwide, particularly in East Asia [16]. In South Korea, Japan, and China, the evidence-based potentiality for safe and effective use of herbal medicine and acupuncture in treatment of acne vulgaris has been suggested [17-27]. However, the current level of evidence is poor due to the small number of good quality studies, small sample size, short duration, and variation in the composition of herbal interventions or acupuncture [28]. In addition, despite common use of a combination of herbal medicine and acupuncture in clinical practice, these studies have rarely demonstrated an interaction between herbal medicine and acupuncture, and have rarely used a waiting list- or western medical treatment group as a control group.

Considering these methodological flaws, we will conduct a trial to assess the interaction effect and efficacy of Keigai-rengyo-to extract (KRTE) and acupuncture using the 2 × 2 factorial design and the feasibility of a large clinical trial. KRTE is an herbal medicine that is often prescribed for treatment of acne; it is approved by the Korea Food and Drug Administration. Females, through the menstrual cycle, can be easily affected by the level of sex hormones, such as estrogen and androgen, relevant to increased sebum production, which play a central role in development of acne; therefore, we will restrict the participants to male patients with acne vulgaris [2,29].

## Method/Design

### Objective

The aim of this study is to investigate the efficacy and interaction of KRTE in male patients with acne vulgaris and evaluate the feasibility of a large clinical trial.

The null hypothesis is as follows: (1) there is no interaction of KRTE and acupuncture treatment on mean percent change of inflammatory acne lesions between baseline and the end of the trial; (2) mean percent change of inflammatory acne lesions between baseline and the end of the trial is equal in both the KRTE treatment group and the waiting list group; (3) mean percent change of inflammatory acne lesions between baseline and the end of the trial is equal in both the acupuncture treatment group and the waiting list group.

### Design

This study is a randomized, 2 × 2 factorial, waiting list controlled, assessor single blinded and single-center pilot trial.

**Figure 1:**
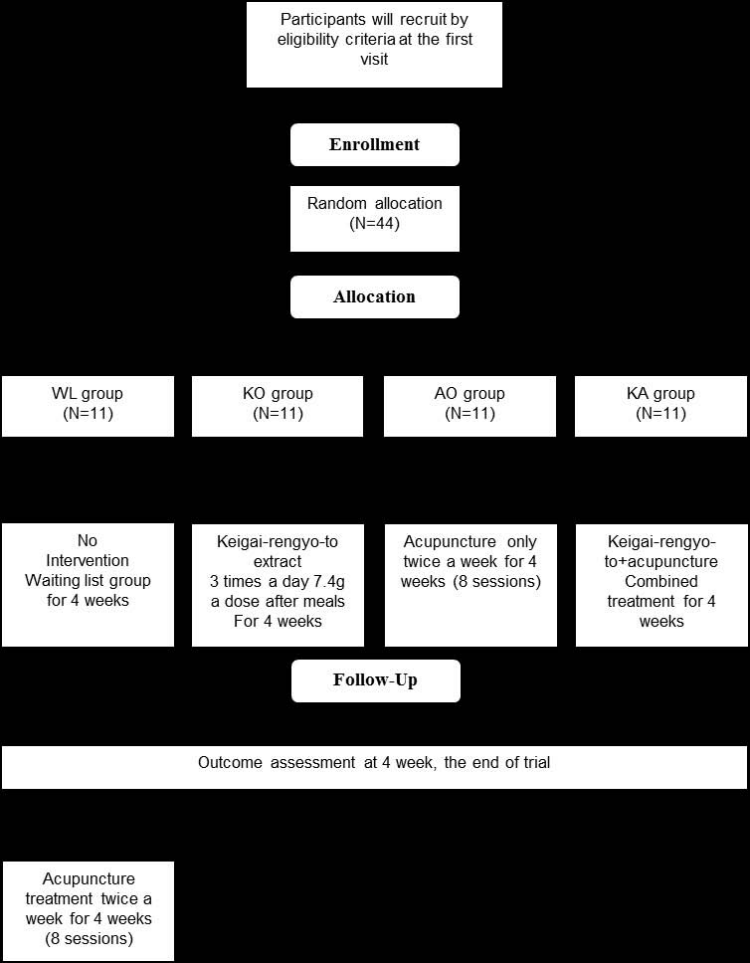
**Flow chart of the study**.

The study will be sequentially conducted as follows: enrollment after screening via inclusion and exclusion criteria, randomization, a treatment period of 4 weeks, and assessment (Figure 1).

### Participants and Eligibility

#### Inclusion criteria

According to the Korean acne grading system [30], patients are divided into 6 grades. Only grade 2 to 4 patients (with papules ≥ 10 or nodules ≤ 20, ± mild ongoing scars), who have had acne for more than 3 months (chronic stage) between the ages of 13 to 35 years, are eligible to participate in this trial.

#### Exclusion criteria

The author will exclude patients outside the normal range on blood tests, including aspartate transaminase (AST), alanine transaminase (ALT), blood urea nitrogen (Bun), creatinine, hemoglobin, and platelet (normal range: 13 ≤ hemoglobin ≤ 17 g/dl, 150000 ≤ platelet ≤ 350000/mm3, aspartate transaminase (AST) < 40 IU/L, alanine transaminase (ALT) < 40 IU/L, 8 ≤blood urea nitrogen (BUN) 23 mg/dl and 0.6 ≤ creatinine ≤ 1.2 mg/dl). Participants are ineligible if they have experienced western medical treatment or traditional medical treatment for acne within 4 weeks prior to recruitment or have keloid acne, corticosteroid acne, infectious skin disease, or internal disease requiring first aid. Participants will also be excluded if they are not willing to not comply with this study protocol. Within 4 weeks after enrollment in this trial, no concomitant acne therapy will be permitted and patients will be instructed to use non-comedogenic makeup or sunscreen.

### Recruitment

Participants will be recruited via internet advertisement posted on the website of the Kyung Hee Medical Center and via ad-posters.

### Randomization and blinding

After enrollment, participants will be randomly assigned to the waiting list (WL), acupuncture only (AO), KRTE only (KO), and KRTE combined with acupuncture treatment (KA) groups. The allocation ratio will be 1:1:1:1 in blocks of 4. Randomization will be performed at a site remote from trial location. Random numbers will be generated by a computerized random-number generator through the block-randomization method of a software program (Excel, Microsoft Office 2007) for sequence generation and we will make two separate database: a "patients" database that lists basic information such as patient name, contact details and so on, and a "randomization" database that holds data on which patients have been registered on trial along with their allocations. The "patients" database will be accessible to any researcher whereas the "randomization" database will be password protected so that it will be accessible only by the principal investigator and a nominated statistician [31]. The assessor, who will not be the acupuncture practitioner and KRTE supplier, and who is blinded to the allocation results until the end of study, will perform the outcome assessment.

### Intervention

#### KRTE

KRTE is approved by the Korea Food and Drug Administration as a prescription in clinical practice for patients with sinusitis, otitis media, and acne. The components of KRTE are presented in Table 1. The authors purchased KRTE from the Han Kook Sin Yak Pharm. Co., which is equipped with a Korean good manufacturing practice (KGMP) facility. Participants in the KO and KA groups will be prescribed KRTE three times a day at a dose of 7.4 g after meals for 4 weeks.

**Table 1:** Components of KRTE (7.4 g/dose)

Scientific Name	Amount (g)
*Schizoneptae Herba*	0.27
*Forsythiae Fructus*	0.34
*Ledebouriellae Radix*	0.65
*Angelicae gigantis Radix*	0.70
*Cnidii Rhizoma*	0.65
*Paeoniae Radix*	0.32
*Bupleuri Radix*	0.59
*Aurantii Fructus*	0.86
*Scutellariae Radix*	0.67
*Gardeniae Fructus*	0.89
*Angelicae dahuricae Radix*	0.35
*Platycodi Radix*	0.51
*Glycyrrhizae Radix*	0.67
**Total**	**7.40**

#### Acupuncture

Certified acupuncture practitioners who have a minimum of 3 years of clinical experience obtained after a 6-year oriental medical college course will perform the acupuncture treatment. They will take a one-day training course for this trial. This course will include the study protocol, methods for acupuncture treatment, and basic information on clinical research. Eight sessions of acupuncture treatment will be performed twice per week for four weeks in patients who will be assigned to the AO and KA groups. Patients will receive acupuncture treatment at 11 classical acupuncture points and/or *ah shi *points that were randomly selected at papules and nodules on the face by the acupuncture practitioner. The following 11 acupuncture points will receive bilateral treatment: ST2 (*Sibai*), ST6 (*Jiache*), ST36 (*Zusanli*), LI20 (*Yingxiang*), LI11 (*Quchi*), PC6 (*Neiguan*), HT8 (*Shaofu*), SP3 (*Taibai*), SP6 (*Sanyinjiao*), SP10 (*Xuehai*), and LR3 (*Taichong*). These points will be treated with 0.25 × 30 mm disposable acupuncture needles (Dongbang Co., Korea). Selection of these acupuncture points was based on a literature review and previous studies of acupuncture treatment of acne vulgaris [20,21] and from the attending doctor's clinical experience and they will be located according to WHO standard Acupuncture Point Locations in the Western Pacific Region [32]. Patients will remain in a supine position for 15 minutes during acupuncture treatment. Papules and nodules will not be artificially extruded.

#### Waiting list group

Participants who will be assigned to the WL group will not receive any acupuncture or KRTE treatment throughout the 4-week post-randomization period. After the end of the study, if participants elect to undergo acupuncture treatment, it will be provided twice weekly for 4 weeks.

### Outcome measures

#### Primary outcome

Primary outcome will be presented by the mean percentage change of inflammatory lesion counts from baseline to the end of the trial [29,33].

#### Secondary outcomes

Secondary outcomes will be shown by the mean percentage change of non-inflammatory lesion counts from baseline to the end of the trial, count of inflammatory and non-inflammatory lesions, the quality-of-life scale (Skindex-29), Visual Analogue Scale (VAS) [score 0 (no symptoms) to 100 (severe symptoms)], and the Investigator Global Assessment [IGA; after evaluation from score 1 (very good) to 5 (very poor) every session, we divided into 'improvement' or 'non-improvement' between baseline and the end of the study]. Skindex 29 will be checked at weeks 0 (baseline) and 4 (end of the trial). VAS and IGA will be checked every session from baseline (just before randomization) and through the 4 weeks after randomization. A Lumix DMC-LX2 digital camera (Panasonic, Osaka, Japan) was used to photograph each patient's face, so that inflammatory and non-inflammatory lesions could be counted at weeks 0 (baseline) and 4 (end of the trial).

### Statistical methods

#### Statistical analysis plan

Statistical analysis will be conducted on an intension-to-treat basis with a 95% confidence interval using SPSS version 12.0 for Windows. Missing values of drop-out participants will be imputed by the last observation carried forward (LOCF) method. Data will be displayed as the mean ± standard deviation (SD) for continuous data or n (%) for categorical data.

#### Baseline data and Outcome data

A two-way analysis of covariance (ANCOVA) test will be used to examine the percentage change of inflammatory and non-inflammatory lesions from baseline to the end of the trial when controlling for baseline and other covariates. A repeated measured ANCOVA test will be performed for evaluation of any significant difference in count of inflammatory and non-inflammatory lesions, Skindex-29 score, and VAS score between baseline and the end of the trial. If there is an interaction between KRTE and acupuncture, only the interaction will be shown. If not, the main effect of KRTE and acupuncture will be reanalyzed and presented. According to 'improvement' or 'no-improvement' of IGA score between baseline and the end of the trial, the difference among the groups will be evaluated using a Chi-square test or Fisher's exact test.

Proportion of drop-outs and compliance with KRTE and questionnaire completion will be calculated and described as n (%). The percentage of subjects in each group will be compared using a Chi-square test or Fisher's exact test.

All adverse events reported during the study will be included in the case report forms; the incidence of adverse events will be calculated. The percentage of subjects with adverse events in each group will be calculated and compared using a Chi-square test or Fisher's exact test.

#### Compliance

KRTE remaining after each session will be quantified in order to enhance medication compliance. Participants whose compliance with KTRE is ≤ 80% of the total dose or who receive fewer than six acupuncture sessions will be considered to have dropped-out.

### Adverse events and monitoring safety

All unexpected adverse events related to KRTE and acupuncture treatment will be reported to the investigator or acupuncture practitioner by participants and written on the individual case report form by the investigator. Safety will be assessed by the reporting of clinical laboratory tests, vital sign measurements, and adverse events. Clinical laboratory tests, including AST/ALT, BUN/creatinine, red blood cell (RBC) count, white blood cell (WBC) count, hemoglobin, hematocrit, mean cell volume (MCV), mean cell hemoglobin (MCH), mean cell hemoglobin concentration (MCHC), number of platelets, and number of differentiated cells will be determined at weeks 0 (baseline) and 4 (end of the trial). Vital signs of each participant will be checked with monitoring of adverse events (pain on the acne lesion or other sites, nausea/vomiting, fatigue, allergic reaction, and any adverse events related to KRTE and acupuncture) after each visit.

### Sample size

This study is a pilot study for evaluation the interaction and efficacy of KRTE and acupuncture in male patients with acne vulgaris and the feasibility of a large clinical trial. Because this trial was designed to be of short duration, lasting four weeks, with the intention of decreasing the drop-out rate, the desired sample size for this pilot study is 44 patients, with 11 for each group, assuming a drop-out rate of 10%.

### Ethics

The study protocol and the written informed consent were approved by the institutional review board (IRB) of the Oriental Medical Hospital at Kyung Hee Medical Center (KOMC IRB 2009-06). Each participant will be notified regarding the study protocol. Written informed consent will be obtained from each patient.

## List of abbreviations

ALT: alanine transaminase; ANOVA: analysis of variance; AO: acupuncture only; AST: aspartate transaminase; BUN: blood urea nitrogen; IGA: investigator global assessment; IRB: institutional review board; KA: KRTE combined with acupuncture treatment; KGMP: Korean good manufacturing practice; KO: KRTE only; KRTE: Keigai-rengyo-to extract; LOCF: last observation carried forward; MCH: mean cell hemoglobin; MCHC: mean cell hemoglobin concentration; MCV: mean cell volume; RBC: red blood cell; RCT: randomized controlled trials; SD: standard WL: waiting list; deviation; TCM: traditional Chinese medicine; VAS: visual analogue scale; WBC: white blood cell.

## Competing interests

The authors declare that they have no competing interests.

## Authors' contributions

KSK participated in the study design, including statistical design, and drafted the manuscript. YBK was the general supervisor for this research and participated in both the study design and critical revision of the manuscript. All authors read and approved the final manuscript.

## References

[B1] Del Rosso JQ Kim G Optimizing use of oral antibiotics in acne vulgaris Dermatol Clin 2009 27 33 42 10.1016/j.clindermatol.2009.09.001 18984366

[B2] Lolis MS Bowe WP Shalita AR Acne and systemic disease Med Clin North Am 2009 93 1161 1181 10.1016/j.mcna.2009.08.008 19932324

[B3] Stern RS The prevalence of acne based on physical examination J Am Acad Dermatol 1992 26 931 935 10.1016/0190-9622(92)70135-3 1535079

[B4] Shamban AT Narurkar VA Multimodal treatment of acne, acne scars and pigmentation Dermatolc Clin 2009 27 459 71 10.1016/j.det.2009.08.010 19850195

[B5] Costa A Lage D Moisés TA Acne and diet: truth or myth? An Bras Dermatol 2010 85 346 353 20676468 10.1590/s0365-05962010000300008

[B6] Arowojolu AO Gallo MF Lopez LM Grimes DA Garner SE Combined oral contraceptive pills for treatment of acne Cochrane Database Syst Rev 2009 8 CD004425 10.1002/14651858.CD004425.pub3 17253506

[B7] Leyden JJ Del Rosso JQ Webster GF Clinical considerations in the treatment of acne vulgaris and other inflammatory skin disorders: a status report Dermatol Clin 2009 27 1 15 18984363 10.1016/j.det.2008.07.008

[B8] Katsambas AD Stefanaki C Cunliffe WJ Guidelines for treating acne Clin Dermatol 2004 22 439 444 10.1016/j.clindermatol.2004.03.002 15556732

[B9] Bettoli V Sarno O Zauli S Borghi A Minghetti S Ricci M Mantovani L Toni G Virgili A What's new in acne? New therapeutic approaches Ann Dermatol Venereol 2010 137 S81 85 10.1016/S0151-9638(10)70033-8 21095503

[B10] Koo J Arain S Traditional Chinese medicine for the treatment of dermatologic disorders Arch Dermatol 1998 134 1388 1393 10.1001/archderm.134.11.1388 9828872

[B11] Yin R Hao F Deng J Yang XC Yan H Investigation of optimal aminolaevulinic acid concentration applied in topical aminolaevulinic acid-photodynamic therapy for treatment of moderate to severe acne: a pilot study in Chinese subjects Br J Dermatol 2010 163 1064 1071 10.1111/j.1365-2133.2010.09860.x 20491770

[B12] Gorpelioglu C Ozol D Sarifakioglu E Influence of isotretinoin on nasal mucociliary clearance and lung function in patients with acne vulgaris Int J Dermatol 2010 49 87 90 10.1111/j.1365-4632.2009.04232.x 20465621

[B13] Geria AN Tajirian AL Kihiczak G Schwartz RA Minocycline-induced skin pigmentation: an update Acta Dermatovenerol Croat 2009 17 123 126 19595269

[B14] Scheinfeld N Bangalore S Facial edema induced by isotretinoin use: a case and a review of the side effects of isotretinoin J Drugs Dermatol 2006 5 467 468 16703787

[B15] McLane J Analysis of common side effects of isotretinoin J Am Acad Dermatol 2001 45 S188 194 10.1067/mjd.2001.113719 11606952

[B16] Koo J Desai R Traditional Chinese medicine in dermatology Dermatol Ther 2003 16 98 105 10.1046/j.1529-8019.2003.01617.x 12919111

[B17] Long X Clinical observation on acne by the treatment of acupuncture combined with traditional Chinese medicine J Liaoning university TCM 2009 11 137 138

[B18] Xu J Liu C Observation on the therapeutic effect of oral taking Chinese medicine plus acupuncture on 62 patients with acne vulgaris Chin J Dermatol Venereol 2009 23 55 56

[B19] Gao CS Chei GB Acupuncture treatment of acne and the use of the mechanism Chin J Aesthetic Med 2009 8 1841 1842

[B20] Lan D Si TR Zhao SL Mao YL Zhang HY Clinical and experimental studies on combination of acupuncture with medicine for treatment of female delayed and persistent acne of different TCM syndrome-types Chin Acupunct Moxibustion 2004 24 379 382

[B21] Xu J Treatment of 80 cases of acne with acupuncture and Chinese drugs J Nanjing TCM university 2005 21 190 191

[B22] Zheng LY Treatment of acne by acupuncture and Chinese herbs Shanghai J Acupunct Moxibustion 2000 19 22 23

[B23] Xu SZ Wang XM Curative effect observation of modified Qingshang Fangfeng decoction match with acupuncture in treatment to acne vulgaris Chin Arch TCM 2007 25 597 598

[B24] Ezequiel P Xu XM Liu GW Experience of integrated acupuncture and medicine in treating acne by professor Liu Gong-wang Tianjin J TCM 2007 24 427 428

[B25] Chan KC A clinical research on combined therapy using acupuncture and herbs to treat common acne in adolescents Guanzhou university Chin Med 2009 1 30

[B26] Dai G Advance in the acupuncture treatment of acne J TCM 1997 17 65 72 10437251

[B27] Nie Y Wang C A survey of treatment of acne by acupuncture J TCM 2008 28 71 74 10.1016/s0254-6272(08)60017-5 18416088

[B28] Morelli V Calmet E Jhingade V Alternative therapies for common dermatologic disorders, part 2 Prim Care 2010 37 285 296 20493337 10.1016/j.pop.2010.02.005

[B29] Koltun W Lucky AW Thiboutot D Niknian M Sampson-Landers C Korner P Marr J Efficacy and safety of 3 mg drospirenone/20 mcg ethinylestradiol oral contraceptive administered in 24/4 regimen in the treatment of acne vulgaris: a randomized, double-blind, placebo-controlled trial Contraception 2008 77 249 56 10.1016/j.contraception.2007.11.003 18342647

[B30] Sung KJ Rho YS Choi EH Oh JJ Lee JH Kim S Kim NI Korean Acne Grading System Korean J Dermatol 2004 42 1241 1247

[B31] Vickers AJ How to randomize J Soc Integr Oncol 2006 4 194 198 10.2310/7200.2006.023 17022927 PMC2596474

[B32] WHO Regional Office for the Western Pacific WHO standard Acupuncture Point Locations in the Western Pacific Region 2008 Manila: World Health Organization

[B33] Leyden J Shalita A Hordinsky M Swinyer L Stanczyk FZ Weber ME Efficacy of a low-dose oral contraceptive containing 20 μg of ethinyl estradiol and 100 μg of levonorgestrel for the treatment of moderate acne: A randomized, placebo-controlled trial J Am Acad Dermatol 2002 47 1 14 10.1067/mjd.2002.122192 12196750

